# Efficacy of donepezil combined with rehabilitation training in the treatment of Alzheimer’s disease

**DOI:** 10.12669/pjms.41.6.11986

**Published:** 2025-06

**Authors:** Yi Yi, Xiaoyan Deng

**Affiliations:** 1 Yi Yi, Department of Neurology, The First Affiliated Hospital of Chongqing Medical and Pharmaceutical College, Chongqing, 400060, P.R. China; 2 Xiaoyan Deng, Department of Neurology, The First Affiliated Hospital of Chongqing Medical and Pharmaceutical College, Chongqing, 400060, P.R. China

**Keywords:** Alzheimer’s disease, Brain-derived neurotrophic factor, Donepezil, Nerve growth factor, Rehabilitation training

## Abstract

**Objective:**

To examine the efficacy of donepezil combined with rehabilitation training for treating Alzheimer’s disease (AD) and its impact on nerve growth factor (NGF) and brain-derived neurotrophic factor (BDNF).

**Methods:**

This single center retrospective analysis was conducted at the First Affiliated Hospital of Chongqing Medical and Pharmaceutical College and included 184 patients with AD treated between January 2022 to May 2024. Of them, 92 patients who received donepezil combined with rehabilitation training (combined group) were matched in a 1:1 ratio with a cohort that received donepezil alone (donepezil group). The treatment efficacy, cognitive function, ability to engage in daily living activity before and after the treatment, serum NGF and BDNF levels, and incidence of adverse reactions were compared between the two groups.

**Results:**

The total efficacy of patients in the combined group was higher than that of the donepezil group (*P*<0.05). After the treatment, mini-mental state examination (MMSE) and activity of daily living (ADL) scores of patients in the combined group were significantly higher than in the donepezil group (*P*<0.05). In contrast, the combined treatment method was associated with considerably lower Alzheimer’s disease assessment scale–cognitive subscale (ADAS-Cog) scores (*P*<0.05). Patients who received the combined treatment had significantly higher serum NGF and BDNF levels than those treated by donepezil alone (*P*<0.05).

**Conclusions:**

Compared with donepezil alone, the combination of donepezil and rehabilitation training is associated with significantly improved therapeutic effects and better serum NGF and BDNF levels.

## INTRODUCTION

With the gradual aging of the world population, the incidence of Alzheimer’s disease (AD) is on the rise.^1^,^2^ AD patients suffer from neurodegeneration, cognitive decline, language disorders, and behavioral abnormalities, which significantly impact their social skills and ability to take care of themselves, often leading to depression and anxiety.^1^–^3^

Studies show that early diagnosis and treatment of AD can slow down the progression of the disease and improve the quality of life.^4^,^5^ Therefore, identifying reliable markers of AD is crucial. Nerve growth factor (NGF) belongs to the category of nerve cell growth and regulation factors, which can participate in nerve cell growth, development, and proliferation. Research has shown that NGF plays an important role in the occurrence and development of AD.^6^,^7^ Brain-derived neurotrophic factor (BDNF) that promotes neurons’ growth, differentiation, and survival has recently emerged as a potential diagnostic biomarker and a therapeutical molecule for AD.^7^ Both NGF and BDNF, therefore, have potential clinical value for staging, subtyping, or precision medicine for AD.^6^–^8^

The pathogenesis of AD is a complex process involving multiple factors. At present, there is no cure for AD, and current therapies mainly focus on temporarily improving symptoms of the disease through drug and non-drug interventions.^9^–^12^ Donepezil is a commonly used medication for treating AD.^10^ However, its effectiveness in treating the declined cognitive function of AD patients is limited.^10^,^11^ Recent studies have suggested the benefits of combining pharmacological methods with non-pharmacological treatment, such as rehabilitation training, as an auxiliary intervention.^10^–^13^ Comprehensive rehabilitation training aims at improving clinical symptoms of AD patients,^6^,^13^ and may lead to a certain degree of improvement in cognitive function.^6^,^12^,^13^

At present, the effect of donepezil combined with rehabilitation training on NGF and BDNF in AD patients is not clear. Therefore, this study aimed to clarify the efficacy of combined donepezil /rehabilitation training regimen in treating AD and its impact on the expression of BDNF and NGF.

## METHODS

This single-center retrospective analysis used the treatment records of 184 patients with AD who received treatment at the First Affiliated Hospital of Chongqing Medical and Pharmaceutical College from January 2022 to May 2024. Of them, 92 patients who received donepezil combined with rehabilitation training (combined group) were matched in a 1:1 ratio with a cohort that received only donepezil treatment (donepezil group), with age being matching variable.

### Ethical Approval:

The ethics committee of our hospital approved this study (Approval Number: 20241126, Date: November 26, 2024).

### Inclusion criteria:

- Meets the diagnostic criteria for AD.^1^
- Age ≥ 60 years old.
- Complete clinical data.

### Exclusion criteria:

- Patients who have taken cognitive-enhancing drugs within four weeks before admission.
- Patients with other diseases that affect cognitive function.
- Patients with a history of alcohol and drug abuse.
- Patients with allergic constitution.
- Patients with severe depression and anxiety.

### Treatment regimen:

### Donepezil

Patients in the donepezil group received donepezil alone. Donepezil (补充药物厂商信息) was orally administered with a standard dose of half a tablet at the initial stage. After one week of treatment, the dosage was increased to one tablet, gradually increasing by half a tablet per week. The dosage was increased to 10mg after one month. The medication was taken continuously for three months.

### Rehabilitation training:

The patient’s medical records after 1-2 days after of admission, including gender, age, course of illness, comorbidities, and years of education, were carefully reviewed. The degree of dementia, cognitive function, mental state, and executive function of patients were assessed, and a personalized rehabilitation training plan was developed and carried out once a day, five times a week, for three consecutive months. The rehabilitation training included:

- Attention training using guessing games, word deletion games, number sequence training, time perception training, and other methods to enhance patients’ attention to specific things; 15 minutes per session, once daily.
- Memory training using directional training methods to enhance patients’ cognitive concepts of time, place, and people; Recalling the past with patients and stimulating their memory of past events; Organizing patients to read news and newspapers every day and increase the frequency of information stimulation; Using auxiliary tools such as laptops, maps, and calendars, to help patients remember their location and search for it; 15 minutes per session, once a day.
- Language function training based on the six principles of Schuell’s aphasia stimulation therapy.^14^ Rehabilitation trainer facilitators selected images, comics, and books to guide patients in repeatedly reading and describing; 60 minutes per session, one day per session.
- Motor function training, including respiratory training, facial muscle training, limb function training, and gait training, 30 minutes per session, once per day.

### Collection indicators:

The following indexes were collected from all patients:

- Clinical characteristics, including sex, age, body mass index (BMI), disease course, education level, smoking, comorbidities, etc.
- Treatment effect estimated using the Alzheimer’s Disease Assessment Scale’s Cognitive Subscale (ADAS-Cog). Efficacy index=the difference in ADAS-Cog before and after the treatment/pre-treatment score x 100%. The treatment effect was categorized based on the changes in effect index values. Significant effect: The therapeutic effect index decreases by ≥ 20%; Effective: the efficacy index decreases by 12% to 19%; Ineffective: the efficacy index decreases by less than 12%; Significant and effective results were included in the total efficacy.
- Cognitive function and daily life activity ability. Cognitive function was evaluated based on the Mini-Mental State Examination (MMSE) and ADAS-Cog. The total score of MMSE is 30 points, with higher scores indicating better cognitive function; the total score of ADAS-Cog is 70, with lower scores indicating better cognitive function. The ability to perform daily activities was evaluated based on the Activity of Daily Living (ADL), with a total score of 100 points. Higher scores indicate better daily life activity ability.
- Serum NGF and BDNF levels were measured by enzyme-linked immunosorbent assay using the AU5800 fully automated biochemical analyzer (Beckman Coulter, USA).
- Relevant adverse reactions, including gastrointestinal symptoms, headache, and rash.

### Statistical Analysis:

All data analyses were conducted using SPSS 25.0 software (IBM Corp, Armonk, NY, USA). The Shapiro-Wilk test was used to evaluate the normality of the evaluation data. Normal distribution data were represented by mean ± standard deviation (SD), independent sample *t*-test was used for inter-group comparison, and paired t-test was used for intra-group comparison before and after the treatment. Non-normally distributed data were represented by median and interquartile range. The Mann-Whitney *U* test was used for inter-group comparisons, and the Wilcoxon signed-rank test was used for intra-group comparisons. The count data were represented by the number of cases and compared using the Chi-square test. PRISM8.0 software (GraphPad, San Diego, USA) was used to plot the changes in ADAS-Cog, MMSE, ADL, NGF, and BDNF before and after treatment. *P*<0.05 was considered statistically significant.

## RESULTS

This study included a total of 184 patients. Among them, there were 98 males and 96 females. The age range is 60-84 years old, with a median age of 68 (63-72) years old. There was no significant difference in clinical characteristics between the two groups of patients (*P*>0.05). Table-I and Table-II shows that the total efficacy of the combination group (96.7%) is significantly higher than that of the donepezil group (85.7%) (*P*<0.05).

**Table-I:** Comparison of clinical characteristics between two groups of patients.

Characteristics	Combined group (n=92)	Donepezil group (n=92)	χ^2^/Z/t	P
Sex (male/female)	51/41	47/45	0.349	0.555
Age (years), M(P25/P75)	68 (63-72)	67 (64-72.5)	-0.314	0.753
BMI (kg/m²), Mean±SD	22.93±2.39	23.1±2.56	-0.473	0.637
Disease course (year), M(P25/P75)	3 (2-3)	2.5 (2-4)	-0.245	0.806
***Educational level, n(%)***				
Junior high school and below	61 (66.3)	67 (72.8)	0.924	0.336
High school and above	31 (33.7)	25 (27.2)		
Smoking (Yes), n(%)	48 (52.2)	40 (43.5)	2.243	0.326
Diabetes (Yes), n(%)	22 (23.9)	29 (31.5)	1.329	0.249
Hypertension (Yes), n(%)	30 (32.6)	24 (26.1)	0.944	0.331
Hyperlipidemia (Yes), n(%)	15 (16.3)	18 (19.6)	0.332	0.564

***Note:*** Body mass index (BMI); Standard deviation (SD).

**Table-II:** Comparison of treatment effects between two groups.

Group	N	Significant effect	Effective	Ineffective	Total effective rate
Combined group	92	54 (58.7)	35 (38.0)	3 (3.3)	89 (96.7)
Donepezil group	92	45 (48.9)	34 (37.0)	13 (14.1)	79 (85.9)
*χ^2^*					7.083
*P*					0.029

**Fig.1:**
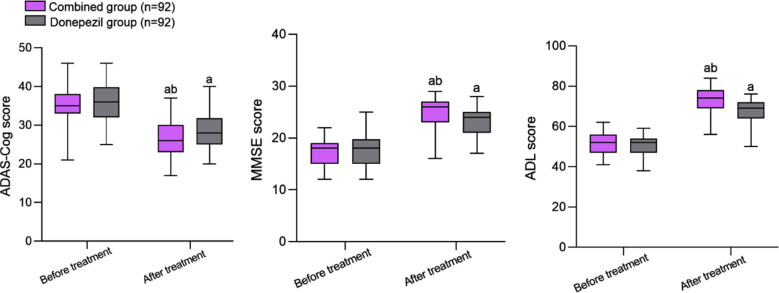
Comparison of cognitive function and activities of daily living between two groups before and after treatment; Compared with before treatment in the same group, ^a^*P*<0.05; Cmpared to donepezil group, ^b^*P*<0.05; Alzheimer’s Disease Assessment Scale Cognitive Assessment (ADAS-Cog); Mini Intelligence State Examination (MMSE); Activity of Daily Living (ADL).

Before treatment, there was no significant difference in ADAS-Cog, MMSE, and ADL scores between the two groups (*P*>0.05); Fig.1. After treatment, the MMSE and ADL scores of both groups increased significantly compared to before treatment, while the ADAS Cog score decreased significantly compared to before treatment; The MMSE and ADL scores of the combined group were significantly higher than those of the donepezil group, while the ADAS Cog score was significantly lower than that of the donepezil group (*P*<0.05).

**Fig.2:**
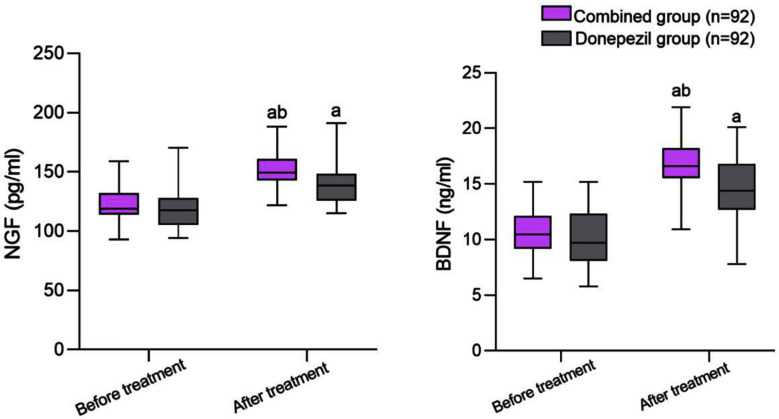
Comparison of serum NGF and BDNF levels between two groups before and after treatment; Compared with before treatment in the same group, ^a^*P*<0.05; Cmpared to donepezil group, ^b^*P*<0.05; Nerve growth factor (NGF); Brain-derived neurotrophic factor (BDNF).

Similarly there was no significant difference in the levels of serum NGF and BDNF between the two groups before treatment (*P*>0.05); Fig.2 After treatment, the levels of serum NGF and BDNF in both groups significantly increased compared to before treatment, and the combined group was significantly higher than the donepezil group (*P*<0.05).There were five cases of adverse reactions in the combination group, accounting for 5.43%; There were three adverse reactions in the donepezil group, accounting for 3.26%; There was no significant difference in the incidence of adverse reactions between the two groups (*P*>0.05) Table-III.

**Table-III:** Comparison of incidence rates of adverse reactions between two groups.

Group	n	Gastrointestinal symptoms	Headache	Rash	Total incidence rate
Combined group	92	2 (2.17)	2 (2.17)	1 (1.09)	5 (5.43)
Donepezil group	92	2 (2.17)	0 (0.00)	1 (1.09)	3 (3.26)
*χ^2^*					0.131
*P*					0.718

## DISCUSSION

The results of this study indicate that in patients with AD, the combination of donepezil and rehabilitation training is associated with a better therapeutic effect than donepezil alone. The combined treatment method is safe and effectively improves cognitive function and recovery of daily living activities. Donepezil is a second-generation cholinergic enzyme inhibitor that, when orally administered, can prevent acetylcholine degradation, enhance nerve conduction function, and improve brain memory and learning abilities.^11^,^12^ It can quickly enter the bloodstream and maintain a stable level to preserve the excitatory function of degenerated neurons and delay the progression of the disease.^10^–^12^ However, numerous studies show that single-drug therapy cannot significantly alleviate the progression of AD.^13^,^15^ In agreement with these reports, the results of this study demonstrated that after treatment, the ADAS-Cog, MMSE, and ADL scores of patients who received the combined treatment were significantly higher than those of patients treated with donepezil monotherapy, further confirming that the effect of oral administration of donepezil alone on daily living function and quality of life of AD patients is limited.

This study showed that a combination of donepezil and rehabilitation training can enhance the treatment effect of AD by effectively improving the memory and cognitive function of patients with AD, enhancing their ability and quality of life, consistent with previous reports.^15^,^16^ Zhang et al.^16^ found that rehabilitation training combined with donepezil can help improve cognitive function and quality of life in AD patients and suggested that this effect may be related to regulating the event-related potential of patients. Wang et al.^17^ also showed that cognitive function can be significantly improved in AD patients through the combination of donepezil and rehabilitation training, and reported that the combined regimen regulated patient event-related potentials and serum levels of BDNF and IGF-1. This study also supports the above research conclusions.

This study found that the adverse reactions in the combination group patients were slightly lower than those treated with donepezil. This observation indicates that rehabilitation training does not impose any additional burden on patients but improves their compliance. In agreement with these conclusions, a study by Kwan et al.^18^ showed that exercise cognitive training effectively promotes cognitive function, reduces physical weakness, and is well tolerated and accepted by elderly people with cognitive weakness. The central nervous system of the human body has plasticity and compensatory properties.

It is plausible that rehabilitation training efficiently stimulates the brain through continuous training, which can enhance the excitability of the nervous system and promote structural reorganization and cell regeneration in damaged areas, thus improving brain function.^16^–^18^ Through purposeful homework activities, patients can develop their understanding of things and concepts of time and space, develop potential cognitive abilities, improve brain intelligence activity, gradually restore life skills., enhance memory ability, attention span, etc. Collectively, these gradual changes are highly beneficial for delaying cognitive decline in AD patients.^17^,^18^

NGF is a neurotrophic factor discovered in 1950 for its properties of promoting growth and survival of peripheral sensory and sympathetic nerve cells.^19^ BDNF is a protein with neurotrophic properties, and it has significant advantages in nerve cell survival and repair, synaptic plasticity, and neuronal cell development.^20^ Studies have also shown that rehabilitation training utilizes the plasticity of the central nervous system, continuously strengthening the ability to transmit stimuli to the brain nerves for system reconstruction, promoting the recovery of neurological function, and thus restoring the normal levels of NGF and BDNF in the body.^21^,^22^ In this study, the combined regimen was associated with a considerably higher total efficacy. The cognitive function and activities of daily living of patients who received rehabilitation training combined with donepezil were markedly better compared to patients on donepezil alone. In addition, this study also found that the levels of NGF and BDNF in the combined group were higher than those in the donepezil group after the treatment. This further supports the feasibility of rehabilitation training combined with donepezil in treating AD from the perspective of neurotrophic factors and confirms that this method can provide a good foundation for slowing disease progression and improving cognitive function recovery, consistent with the existing research.^23^-^26^ Rehabilitation training can stimulate brain injury repair and compensatory function from multiple aspects, thereby achieving better therapeutic effects.^27^

Taken together, the results of this study can provide practical reference and guidance for the clinical treatment of AD, promote the diversification of treatment, and improve the outcome of AD patients.

### Limitations

It is a single-center retrospective study with a small sample size. Both groups of patients were not randomly assigned, potentially resulting in selection bias. Secondly, only patients over 60 years old were included in this study. Thirdly, this study did not conduct follow-up observations on patients or explore changes in cerebral blood flow status after the treatment. Fourthly, in this study, patients who did not experience worsening of their condition or adverse reactions were allowed to continue using donepezil combined with rehabilitation training for intervention treatment under clinical doctors’ guidance after three months of treatment. Finally, the impact of combination therapy with donepezil and rehabilitation training on the long-term functional recovery of patients was not analyzed. Further prospective, higher-quality studies with large sample sizes, wider demographics, and different ages are needed to validate these results.

## CONCLUSION

Combining donepezil and rehabilitation training in treating AD is associated with improved cognitive function, ability to engage in daily living activity, and better overall treatment effectiveness than donepezil monotherapy. The combined method more effectively improves serum NGF and BDNF levels without causing additional adverse reactions.

### Authors’ contributions:

**YY:** Study design, literature search and manuscript writing.

**YY** and **XD:** Data collection, data analysis and interpretation. Critical review.

**YY:** Manuscript revision and validation, critical analysis.

All authors have read and approved the final manuscript and are responsible for the integrity of the study.
